# Delivery of screening and brief intervention for unhealthy alcohol use in an urban academic Federally Qualified Health Center

**DOI:** 10.1186/s13722-017-0100-2

**Published:** 2017-12-07

**Authors:** Marcus A. Bachhuber, Megan A. O’Grady, Henry Chung, Charles J. Neighbors, Joseph DeLuca, Elenita M. D’Aloia, Arelis Diaz, Chinazo O. Cunningham

**Affiliations:** 1 Division of General Internal Medicine, Department of Medicine, Montefiore Medical Center/Albert Einstein College of Medicine, 3300 Kossuth Ave, Bronx, NY 10467 USA; 2 Department of Health and Treatment Research and Analysis, The National Center on Addiction and Substance Abuse at Columbia University, 633 Third Avenue, 19th Floor, New York, NY 10017 USA; 3 Montefiore Care Management, Montefiore Medical Center, 200 Corporate Boulevard South, Yonkers, NY 10701 USA; 4 Montefiore Medical Group, Montefiore Medical Center, 305 E 161st St, Bronx, NY 10451 USA

**Keywords:** Alcohol use disorder, Unhealthy alcohol use, Screening and brief intervention, Primary care, Screening

## Abstract

**Background:**

Screening and brief intervention (SBI) for unhealthy drinking has not been widely implemented in primary care partly due to reliance on physicians to perform it.

**Methods:**

We implemented a model of nursing staff-delivered SBI for unhealthy drinking for adult patients receiving primary care at an academically-affiliated Federally Qualified Health Center in the Bronx, NY. Our model consisted of nursing staff screening all patients with the alcohol use disorders identification test consumption questions (AUDIT-C) and, if screening positive, providing BI or referral to specialty services. We developed a clinical decision support tool integrated into the electronic health record to guide nursing staff and record SBI provision. To evaluate this model, we determined overall SBI delivery to patients and factors associated with receiving SBI.

**Results:**

Between October 2013 and September 2014, 9119 unique adult patients made 24,285 visits. Patients were majority women (67.5%) and Hispanic/Latino (54.5%). Overall, 46.2% were screened, with 19.0–35.8% of eligible patients screened in each month. Increasing age (OR: 0.82 [95% CI 0.80–0.85] for a 10-year increase), female sex (OR: 0.83 [95% CI 0.77–0.91]), and chronic conditions like hypertension (OR: 0.62 [95% CI 0.56–0.70]) and diabetes (OR: 0.66 [95% CI 0.58–0.75]), among others, were associated with a lower odds of being screened. Of all patients screened, 225 (5.3%) screened positive and of those patients, 122 (54.2%) received a BI. Patients with higher AUDIT-C scores were more likely to receive a BI (OR: 1.24 [95% CI 1.04–1.47] for a 1-point increase) and non-English speaking patients were less likely to receive a BI than those who spoke English (OR: 0.42 [95% CI 0.18–0.97]).

**Conclusions:**

Our model of SBI resulted in screening of nearly half of all eligible patients and BI provision to over half of those screening positive. Future efforts to improve SBI delivery should focus on groups such as older adults, women, and those with chronic medical conditions.

## Background

Unhealthy alcohol use, defined as drinking at a level that can lead to negative health consequences [1], is a leading cause of preventable morbidity and mortality in the US [2]. Screening and brief intervention for unhealthy drinking (SBI) can reduce self-reported drinking in primary care settings [3]. Despite the US Preventive Services Task Force recommendation that all adults receive SBI [4], it is uncommonly provided, resulting in missed opportunities to improve outcomes [5]. In a recent national study, while 71.1% of respondents reported that their doctor asked about alcohol use (not necessarily with standardized validated screening tools), only 4.4% of those with heavy episodic (i.e., binge) drinking reported being advised to cut back [6]. This gap between evidence and practice is likely the result of several barriers, including competing clinical priorities, staff training and knowledge, and organizational factors [7].

In settings that deliver SBI, physicians have traditionally provided it during routine clinical care; however, physicians’ competing priorities are a barrier to broader implementation of SBI [8–10]. In line with efforts to move toward team-based primary care, SBI delivery by non-physician providers—often nursing staff—is feasible and efficacious [11]. Two implementation trials in primary care settings found that non-physician-delivered SBI resulted in a higher percentage of patients screened than physician-delivered SBI (24 vs. 19% and 51 vs. 9%) [12, 13]. One trial found that non-physician-delivered SBI resulted in higher rates of BI provision among patients screening positive than physician-delivered SBI (73.1 vs. 57.1%) [12] and one found the opposite (3.4 vs. 44.0%) [13]. Despite intensive efforts, both studies also revealed that a significant percentage of patients did not receive SBI.

While many non-physician providers receive less training and feel less comfortable addressing unhealthy alcohol use than physicians [14], clinical decision support (CDS) tools—electronic health record (EHR)-based tools designed to aid clinicians in making diagnostic and treatment decisions—can mitigate these challenges and have been widely used to promote guideline-concordant care [15]. To increase provision of alcohol screening, the Veterans Health Administration (VA) successfully used a CDS tool (i.e., a clinical reminder) in combination with provider training and a national performance measure, resulting in very high rates of screening (93%) [16].

On an organizational level, incorporating new practices such as SBI into existing clinical systems can be challenging, but some strategies can improve this process. For example, piloting with teams including front-line clinicians, leadership, and administration can help ensure that everyone affected by a new practice has an opportunity to provide input and feedback [17]. Identifying and working with clinical champions can also promote implementation; specifically, physician and nursing champions can help to serve as opinion leaders promoting SBI [18].

With the goal of improving the delivery of evidence-based preventive care, we implemented routine SBI for unhealthy drinking at an adult medicine practice. To build on findings from previous research and utilize practices shown to enhance the success of implementation, our model consisted of nursing staff-delivered SBI with an SBI-focused CDS tool that was piloted and promoted by a physician and nurse champion. To target future improvements, we also conducted an evaluation of this model to determine overall SBI delivery and factors associated with receiving SBI.

## Methods

### Study setting and population

The study setting is a Federally Qualified Health Center affiliated with our academic institution in the Bronx, NY. It is a multispecialty practice with internal medicine, pediatrics, obstetrics/gynecology, dermatology, podiatry, and dentistry, with onsite behavioral health, laboratory, and radiology services. We implemented SBI in the internal medicine practice which includes 11 Licensed Practical Nurses, two Patient Care Technicians (similar to medical assistants), one Registered Nurse, 15 attending physicians, and 30 resident physicians. Over 9000 unique adult patients attend about 25,000 visits per year.

### Model of care

Our model of SBI for unhealthy drinking consisted of annual screening by nursing staff followed by delivery of a BI for patients with unhealthy alcohol use. Patients eligible for screening included all adult patients seen for any type of visit. Nursing staff performed screening prior to patients’ visits with physicians, at the same time they measured vital signs and weight. If a BI was warranted, nursing staff performed it immediately after screening, before physician encounters.

To improve delivery of SBI, we developed a CDS tool integrated into our EHR (Centricity Electronic Medical Record version 9.8) consisting of three components: (1) a clinical reminder for screening, (2) screening questions and fields to record answers, and (3) prompts for BI and a field to record delivery. For the clinical reminder, we integrated it into the note in the same location as reminders for other preventive services (e.g., colorectal cancer screening). The reminder was not a “hard stop” and could be ignored; if ignored, it would appear again at patients’ next visit. If screening was performed, the reminder would not appear for 1 year. Clicking on the reminder activated the screening questions. While our model of SBI relied on nursing staff, the clinical reminder was visible to physicians and could be completed by them; however, anecdotally, this was rare due to competing clinical priorities.

**Fig. 1:**
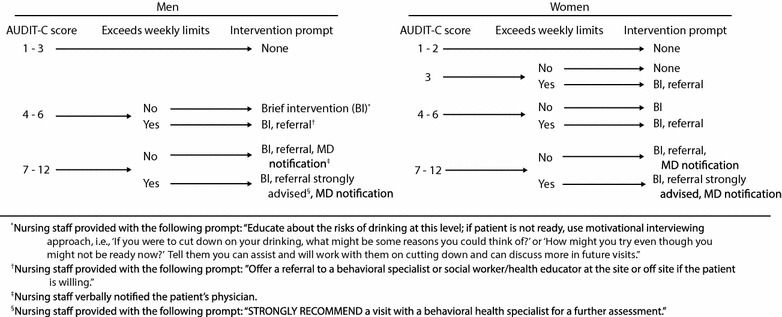
Algorithm for screening and brief intervention for unhealthy drinking in an urban academic Federally Qualified Health Center

To screen, nursing staff used the alcohol use disorders identification test consumption questions (AUDIT-C) [19, 20]: (1) “How often did you have a drink containing alcohol in the past year?”; (2) “How many drinks did you have on a typical day when you were drinking in the past year?”; and (3) “How often did you have six or more drinks on one occasion in the past year?” Each item is scored from 0 to 4 points (total possible score = 0–12 points). For women scoring ≥ 3 points, a question appeared asking, “Does the drinking level exceed 7 drinks per week?”; for men scoring ≥ 4 points, the question read “Does the drinking level exceed 14 drinks per week?” [21]. We included questions about exceeding weekly limits because the AUDIT-C does not map precisely on to recommended drinking limits. Based on the patient’s score, nursing staff were prompted to provide a verbal BI, referral to behavioral health staff (e.g., social work), a verbal notification to the patient’s physician, or combinations of the above. The SBI algorithm and the specific BI components delivered by nursing staff at each AUDIT-C score are shown in Fig. 1.

Prior to implementation, we piloted the CDS tool and screening procedure with a nurse champion. The purpose of this step was to test the CDS tool, determine if nursing staff would be able to screen patients while collecting vital signs and chief complaint, determine the time it took to screen, and gauge patients’ responses. In addition to the nurse champion, an attending physician champion specializing in addiction medicine also took a lead role in testing and suggesting changes to the CDS tool. Furthermore, we involved the medical director and other administrative and clinical staff at the clinic throughout the piloting phase to ensure that all staff affected by this new practice could give feedback. After the pilot, we trained the 12 members of the nursing staff as well as attending and resident physicians in separate 1-h meetings. These meetings covered the rationale for screening for unhealthy alcohol use, best practices in administering the AUDIT-C, the evidence supporting the benefit of BI, the overall framework of BI and best practices in administering BI, and the practicalities of SBI at the clinic including the workflow.

### Evaluation and data collection

To evaluate implementation, we extracted data on all adult medicine patients seen within 1 year after implementation of routine SBI (October 2013–September 2014). From the EHR, we extracted patient data (e.g., demographics), visit data (e.g., visit diagnoses), and results of the screener (e.g., the AUDIT-C score and whether a BI was performed).

### Main outcome measures

Our main outcomes were documentation of screening, screening positive for unhealthy drinking, and documentation of BI provision. While nurses were instructed to provide BIs according to the algorithm (Fig. 1), we were unable to extract EHR data from the question about exceeding weekly limits; therefore, in the evaluation we considered an AUDIT-C score of ≥ 4 as a positive screen for both men and women. Of all women, 93 received an AUDIT-C score of 3 points and some of those would have ultimately been considered a positive screen at their visit (i.e., if they also exceeded weekly limits) and been eligible to receive a BI.

### Visit and patient factors

From EHR data, we coded several visit and patient factor variables. For visits, we collected the type of visit (e.g., new patient, established patient, or urgent care/walk-in) and the month in which the visit occurred. New patient visits were defined as the patient’s first ever visit to the clinic or the patient’s first visit with a new provider (i.e., transition of care). For patient sociodemographics, we collected age, sex, race/ethnicity, and preferred language. Finally, we identified patients’ chronic medical conditions using visit diagnoses (i.e., diagnoses coded as actively addressed) grouped into clinically meaningful categories using established coding schemes [22].

### Statistical analysis

First, we summarized patient characteristics using descriptive statistics. Next, we determined the number and percentage of patients screened overall and in each month, the number and percentage screening positive, and the number and percentage of patients screening positive who received a BI.

We then determined visit and patient factors associated with being screened, screening positive, and receiving a BI if screening positive. We created three separate multivariable logistic regression models. In the first model, to determine visit and patient factors associated with being screened, the dependent variable was whether the patient was screened. To account for the possibility of patients having multiple visits over the study period, we used person-visit as the unit of analysis, using a generalized estimating equations model accounting for clustering at the patient level and producing robust standard errors. We included visit type (new patient, established patient, or urgent care/walk-in), visit month, and all patient factors as independent variables. In the second model, to determine patient factors associated with screening positive for unhealthy drinking, the dependent variable was the presence of a positive screen among those who were screened. Because of a reduced sample size and collinearity, we removed race/ethnicity from the model and combined several categories for language, visit type, and chronic conditions. In the third model, to determine patient factors associated with receiving a BI among those screening positive, the dependent variable was the documentation of BI among those screening positive. The independent variables were similar to the previous model with the addition of AUDIT-C score. For the second and third model, we analyzed only the visit at which screening was performed (i.e., only one visit per patient); therefore, the unit of analysis in these models is the patient.

We evaluated the associations of patient factors with outcomes in terms of odds ratios and 95% confidence intervals (95% CIs). We also used predictive margins to describe each factor’s association with outcomes in terms of the difference in probability of the outcome (in percentage points) compared to the referent group, after adjusting for all other factors. We conducted all analyses with SAS 9.4 (Cary, NC) and Stata 13.1 (College Station, TX).

## Results

Between October 2013 and September 2014, 9119 adult patients attended one or more visit. The mean number of visits per patient was 2.7 (range 1–32). Patients were majority women (67.5%) and Hispanic/Latino (54.5%; Table 1). Most patients preferred English (71.5%), followed by Spanish (25.4%). The most common chronic conditions among patients were hypertension (26.5%), diabetes (14.8%), and depression (13.1%).

**Table 1:** Demographic and clinical characteristics of adult medicine patients of an urban academic Federally Qualified Health Center during implementation of a screening and brief intervention initiative (n = 9119)

Characteristic	n (%)
Age, median (IQR)	48.8 (33.7, 60.9)
Female sex	6153 (67.5)
Race/ethnicity	
Black, non-Hispanic	945 (10.4)
Hispanic, of any race	4965 (54.5)
Any other or undetermined race^a^	3209 (35.2)
Language	
English	6518 (71.5)
Spanish	2316 (25.4)
French	285 (3.1)
Chronic conditions	
Hypertension	2420 (26.5)
Diabetes	1345 (14.8)
Congestive heart failure	162 (1.8)
Chronic kidney disease	305 (3.3)
HIV	255 (2.8)
Hepatitis C virus	113 (1.2)
Depression	1194 (13.1)
Opioid or cocaine use disorder	460 (5.0)

^a^Includes White, Asian/Pacific Islander, Native American/Alaskan Native, or more than one race

**Fig. 2:**
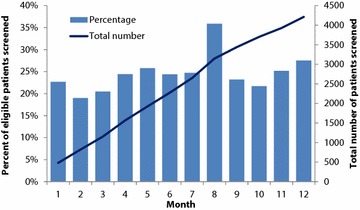
Percent and cumulative number of eligible adult medicine patients screened for unhealthy alcohol use, by month, in an urban academic Federally Qualified Health Center. Light blue bars represent percentage of patients screened in each month of the study period (left axis). Dark blue line represents the cumulative number of patients screened (right axis)

Overall, 4212 (46.2%) patients were screened with 19.0–35.8% of eligible patients screened in each month (Fig. 2). Of all patients screened, 2767 (65.6%) were screened on their first visit during the study period. Increasing age (10-year increment, OR: 0.82 [95% CI 0.80–0.85]), female sex (OR: 0.83 [95% CI 0.77–0.91]), and chronic conditions such as hypertension (OR: 0.62 [95% CI 0.56–0.70]) and diabetes (OR: 0.66 [95% CI 0.58–0.75]), among others, were associated with a lower odds of being screened (Table 2). Screening was also less likely at established patient visits (OR: 0.22 [95% CI 0.20–0.24]) and urgent care/walk-in visits (OR: 0.12 [95% CI 0.11–0.14]) compared to new patient visits. The odds of screening increased in each month during the study period (OR: 1.03 [95% CI 1.02–1.04]).

**Table 2:** Screening and factors associated with screening for unhealthy alcohol use among adult medicine patients at an urban academic Federally Qualified Health Center (n = 24,285 visits among 9119 patients)

Characteristic	Screened, n (%)^a^	Adjusted odds ratio, 95% CI	*p*	Adjusted difference in percent screened, % (95% CI)^b^
All	4212 (46.2)	–	–	–
Age^c^		0.82 (0.80–0.85)	< 0.001	− 2.5 (− 2.9 to − 2.1)
18–29 years	1022 (58.8)			
30–49	1493 (48.7)			
50–64	1079 (41.8)			
≥ 65	618 (35.6)			
Sex				
Male	1479 (49.9)	Ref.	< 0.001	Ref.
Female	2733 (44.4)	0.83 (0.77–0.91)		− 2.4 (− 3.5 to − 1.3)
Race				
Black, non-Hispanic	420 (44.4)	Ref.		Ref.
Hispanic, of any race	2298 (46.6)	0.95 (0.83–1.10)	0.53	− 6.0 (− 2.5 to 1.3)
Any other or undetermined race^d^	1494 (46.3)	1.00 (0.87–1.15)	0.99	0.02 (− 1.8–1.8)
Language				
English	3450 (47.1)	Ref.		Ref.
Spanish	1301 (43.8)	1.02 (0.92–1.14)	0.68	0.3 (− 1.1 to 1.7)
French	156 (45.3)	0.94 (0.75–1.18)	0.61	− 0.75 (− 3.6 to 2.1)
Visit type				
New patient	–	Ref.		Ref.
Established patient	–	0.22 (0.20–0.24)	< 0.001	− 28.3 (− 0.30.3 to − 26.4)
Urgent care or walk-in	–	0.12 (0.11–0.14)	< 0.001	− 34.3 (− 36.3 to − 32.3)
Visit month	–	1.03 (1.02–1.04)	< 0.001	0.4 (0.3–0.6)
Chronic conditions				
Hypertension				
No	3421 (51.1)	Ref.		Ref.
Yes	791 (32.7)	0.62 (0.56–0.70)	< 0.001	− 5.9 (− 7.3 to − 4.6)
Diabetes				
No	3790 (48.8)	Ref.		Ref.
Yes	422 (31.4)	0.66 (0.58–0.75)	< 0.001	− 5.1 (− 6.6 to − 3.7)
Congestive heart failure				
No	4152 (46.4)	Ref.		Ref.
Yes	60 (37.0)	1.08 (0.80–1.47)	0.60	1.1 (− 3.0 to 5.1)
Chronic kidney disease				
No	4114 (46.7)	Ref.		Ref.
Yes	98 (32.1)	0.96 (0.75–1.22)	0.74	− 0.5 (− 3.6 to 2.5)
HIV				
No	4135 (46.7)	Ref.		Ref.
Yes	77 (30.2)	0.36 (0.28–0.45)	< 0.001	− 10.5 (− 12.3 to − 8.6)
Hepatitis C virus				
No	4173 (46.3)	Ref.		Ref.
Yes	39 (34.5)	0.64 (0.44–0.93)	0.02	− 5.1 (− 8.9 to − 1.3)
Depression				
No	3729 (47.1)	Ref.		Ref.
Yes	483 (40.5)	0.75 (0.66–0.84)	< 0.001	− 3.6 (− 5.0 to − 2.3)
Opioid or cocaine use disorder				
No	3983 (46.0)	Ref.		Ref.
Yes	229 (49.8)	0.76 (0.64–0.91)	0.002	− 3.3 (− 5.3 to − 1.3)

^a^Percentages calculated at the patient level (i.e., the percent of male patients). Regression models estimated using patient-visit as the unit of analysis

^b^Refers to the absolute difference in the probability of being screened, adjusting for all variables

^c^Age categories used for descriptive statistics, age used as a continuous variable in regression models with a 1-unit change representing 10 years

^d^Includes White, Asian/Pacific Islander, Native American/Alaskan Native, or more than one race

Of all screened patients, 225 (5.3%) screened positive (≥ 4 points on the AUDIT-C; Table 3). Increasing age (OR: 0.79 [95% CI 0.71–0.87]), female sex (OR: 0.32 [95% CI 0.24–0.42]), and non-English language (OR: 0.50 [95% CI 0.33–0.75]) were associated with a lower odds of screening positive. Patients screened during established or urgent care visits were also less likely to screen positive than new patients (OR: 0.53 [95% CI 0.40–0.71]).

**Table 3:** Factors associated with screening positive for unhealthy drinking among adult medicine patients who were screened at an urban academic Federally Qualified Health Center (n = 4212 patients)

Characteristic	Screened positive, n (%)	Adjusted odds ratio, 95% CI	*p*	Adjusted difference in percent screened, % (95% CI)^a^
All	225 (5.3)	–	–	–
Age^b^		0.79 (0.71–0.87)	< 0.001	− 1.2 (− 1.7 to − 6.8)
18–29 years	78 (7.6)			
30–49	93 (6.2)			
50–64	46 (4.3)			
≥ 65	8 (1.3)			
Sex				
Male	139 (9.4)	Ref.		Ref.
Female	86 (3.2)	0.32 (0.24–0.42)	< 0.001	− 5.9 (− 7.5 to − 4.4)
Language				
English	195 (6.4)	Ref.		Ref.
Spanish or French	30 (2.6)	0.50 (0.33–0.75)	0.001	− 2.9 (− 4.2 to − 1.5)
Visit type				
New patient	148 (7.7)	Ref.		Ref.
Established patient or urgent care/walk-in	77 (3.4)	0.53 (0.40–0.71)	< 0.001	− 3.0 (− 4.4 to − 1.6)
Chronic medical or mental health condition				
No	154 (5.7)	Ref.		Ref.
Yes	71 (4.7)	1.24 (0.91–1.70)	0.18	1.1 (− 0.05 to 2.7)

Defined as an AUDIT-C score of ≥ 4

^a^Refers to the absolute difference in the probability of screening positive, adjusting for all variables

^b^Age categories used for descriptive statistics, age used as a continuous variable in regression models with a 1-unit change representing 10 years

Of all patients screening positive, 122 (54.2%) received a BI (Table 4). Non-English speaking patients were less likely to receive a BI than those who spoke English (OR: 0.40 [95% CI 0.17–0.91]). Patients with higher AUDIT-C scores were more likely to receive a BI (OR: 1.24 [95% CI 1.04–1.47] for a 1-point increase in AUDIT-C).

**Table 4:** Delivery of brief intervention and factors associated with delivery among adult medicine patients who screened positive for unhealthy drinking at an urban academic Federally Qualified Health Center (n = 225 patients)

Characteristic	Received brief intervention, n (%)	Adjusted odds ratio, 95% CI	*p*	Adjusted difference in percent screened, % (95% CI)^a^
All	122 (54.2)	–	–	–
Age^b^		1.07 (0.87–1.32)	0.53	1.6 (− 3.4 to 6.6)
18–29 years	46 (59.0)			
30–49	43 (46.2)			
50–64	27 (58.7)			
≥ 65	6 (75.0)			
Sex				
Male	73 (52.5)	Ref.		Ref.
Female	49 (57.0)	1.24 (0.69–2.21)	0.47	5.0 (− 8.6 to 18.5)
Language				
English	111 (56.9)	Ref.		Ref.
Spanish or French	11 (36.7)	0.42 (0.18–0.97)	0.04	− 20.3 (− 38.9 to − 1.7)
Visit type				
New patient	84 (56.8)	Ref.		Ref.
Established patient or urgent care/walk-in	38 (49.4)	0.75 (0.42–1.32)	0.33	− 6.7 (− 19.9 to 6.6)
AUDIT-C score	–	1.24 (1.04–1.47)	0.01	5.1 (1.2–8.9)
Chronic medical or mental health condition				
No	82 (53.3)	Ref.		Ref.
Yes	40 (56.3)	1.01 (0.56–1.84)	0.96	0.3 (− 13.6 to 14.3)

Defined as an AUDIT-C score of ≥ 4

^a^Refers to the absolute difference in the probability of receiving a brief intervention, adjusting for all variables

^b^Age categories used for descriptive statistics, age used as a continuous variable in regression models with a 1-unit change representing 10 years

## Discussion

Using a model of nursing staff-delivered SBI with an integrated CDS tool, our adult medicine practice screened almost half of all patients and provided a BI to over half of patients who screened positive. Monthly rates of screening increased modestly over the 1-year study period. Our study is among the few published studies reporting SBI rates and factors associated with receipt of SBI, in which SBI is integrated into routine care and delivered by nursing staff that are not grant funded [16]. As most clinical settings would not have additional grant funding available for SBI implementation, our findings are significant by showing that SBI can be integrated into routine care in an urban safety net setting without additional funding by using team-based models along with other implementation facilitators such as clinical champions, EHR CDS tools, and getting feedback and buy-in among all team members of the clinic.

While significant room for improvement remains, the rates of screening and BI we achieved are in the range of previous studies [23]. However, our screening rates (46.2%) were lower than rates reported in the VA (93%) [16]. This may be due to several factors in the VA system such as high levels of clinician comfort with clinical reminders and use of a system-wide alcohol screening performance measure to provide feedback to clinicians. The rates of BI we achieved (54.2%) were higher than in the non-physician provider arm of one implementation trial (3.4%) [13]. In that trial, provision of BI was dependent on availability of additional staff and rooms, compared to our model where BI was performed within routine patient flow.

We identified specific visit and patient characteristics that were associated with lower odds of being screened, including older age, female sex, and chronic medical conditions. Lower rates of screening among older adults and women may be related to assumptions or inferences about patient responses without asking screening questions verbatim [24]. Lower rates of screening among those with chronic medical conditions may be due to competing clinical priorities. For example, nursing staff routinely measure blood capillary glucose for patients with diabetes. For patients with depression, nursing staff also routinely administer depression questionnaires and record them in the EHR. As unhealthy alcohol use affects all demographics and can exacerbate many chronic medical conditions [25–29], reinforcement of the importance of SBI for all patients is essential. Furthermore, adjusting clinical processes to allow for extra time (e.g., reducing the frequency of blood capillary glucose measurements) may be necessary to improve SBI delivery among those with chronic medical conditions. Beyond these factors, qualitative research with nursing staff could help identify other areas where SBI delivery can be improved.

In our evaluation, 5.3% of patients screened had a positive screen. While we had to modify our definition of a positive screen due to technical limitations (to ≥ 4 AUDIT-C points for men and women), this rate of positive screens is at the low end of what we would expect based on community surveys. In a 2014 survey, 4.6% of Bronx adults reported heavy drinking—defined in that survey as an average of > 2 drinks per day for men and > 1 drink per day for women; 14.6% reported binge drinking—defined as ≥ 5 drinks on one occasion for men and ≥ 4 drinks on one occasion for women in the past 30 days [30]. As our CDS tool only included screening questions in English, one potential explanation for a relatively low rate of positive screens may be language. While non-English speakers were screened at similar rates as English speakers, non-English language was associated with a lower odds of screening positive, suggesting that nursing staff were able to translate screening questions ad hoc, but in doing so, they may have lost sensitivity. Further study is needed to compare the sensitivity of our routine screening to a reference standard. In VA facilities, for example, routine screening has had an unexpectedly low sensitivity [31, 32].

For BI, a higher AUDIT-C score was associated with a higher odds of receiving the intervention, suggesting that, while nursing staff were not providing a BI to all patients, they were prioritizing those with higher AUDIT-C scores. Non-English language was the only factor associated with a lower odds of receiving the intervention. This finding is similar to the VA, where Hispanic/Latino patients are less likely to receive services for unhealthy alcohol use than white and black patients after screening positive [33]. Specific to our clinic, while most nursing staff are bilingual English and Spanish or English and French speakers, they often care for patients who speak a different language. Although telephone interpreters are available on demand, this may create an unreasonable time delay; future work should examine alternatives such as interactive voice response or video-based BI for language-discordant encounters [34, 35]. More generally, future evaluations of this model of routine SBI should evaluate fidelity of nursing staff to the recommended BI framework and, most importantly, effects on clinical outcomes such as self-reported drinking and disease control.

This study has limitations. First, because of EHR limitations, we could not identify individual nursing staff or physicians providing SBI. As provider factors may be more important than visit and patient factors in determining which patients receive SBI, future research is needed to assess providers’ impact. We also could not extract results for the question asking about weekly drinking limits which required us to use a nonstandard threshold for unhealthy drinking for women. This limitation of our data may have led to biased estimates of factors associated with screening positive and factors associated with receiving a BI; the bias could be in either direction. While BI provision was documented in the EHR, we did not assess fidelity to recommended BI framework. Our ability to examine the impact of race/ethnicity on screening and receipt of a BI was limited by the large number of patients with this variable recorded as “undetermined”. Because our goal was to deliver SBI, we did not have a comparison group. Furthermore, SBI was not routinely provided prior to our study and the AUDIT-C questions were not available in the EHR, which also makes historical controls unavailable. Finally, during the study period, referrals to behavioral health providers were paper-based and therefore we cannot determine the number of patients referred after screening positive.

## Conclusions

In a Bronx Federally Qualified Health Center without dedicated grant-funded personnel, we integrated a model of nursing staff-delivered routine SBI for unhealthy drinking into primary care. Almost half of the patients presenting for one or more visit were screened, and of those who screened positive, over half received a BI. Patient characteristics including older age, female sex, and chronic illnesses were associated with lower odds of screening; non-English language was associated with lower odds of receiving a BI. While integrating SBI into routine primary care by nursing staff can lead to moderate screening rates, it is important to ensure that SBI is delivered to patients who could clinically benefit most (e.g., those with chronic diseases impacted by unhealthy alcohol use). Health care facilities need to continue to integrate models of SBI that are comprehensively delivered to patients in routine primary care.

## Abbreviations

SBI: screening and brief intervention
BI: brief intervention
EHR: electronic health record
CDS: clinical decision support
AUDIT-C: alcohol use disorders identification test consumption questions
